# *StarPep Toolbox*: an open-source software to assist chemical space analysis of bioactive peptides and their functions using complex networks

**DOI:** 10.1093/bioinformatics/btad506

**Published:** 2023-08-21

**Authors:** Longendri Aguilera-Mendoza, Sebastián Ayala-Ruano, Felix Martinez-Rios, Edgar Chavez, César R García-Jacas, Carlos A Brizuela, Yovani Marrero-Ponce

**Affiliations:** Grupo de Medicina Molecular y Translacional (MeM&T), Facultad de Medicina, Universidad San Francisco de Quito (USFQ), Quito, Ecuador; Departamento de Ciencias de la Computación, Centro de Investigación Científica y de Educación Superior de Ensenada (CICESE), Ensenada, Baja California 22860, México; Grupo de Medicina Molecular y Translacional (MeM&T), Facultad de Medicina, Universidad San Francisco de Quito (USFQ), Quito, Ecuador; Facultad de Ingeniería, Universidad Panamericana, CDMX, Benito Juárez 03920, México; Departamento de Ciencias de la Computación, Centro de Investigación Científica y de Educación Superior de Ensenada (CICESE), Ensenada, Baja California 22860, México; Departamento de Ciencias de la Computación, Centro de Investigación Científica y de Educación Superior de Ensenada (CICESE), Ensenada, Baja California 22860, México; Cátedras CONAHCYT - Departamento de Ciencias de la Computación, Centro de Investigación Científica y de Educación Superior de Ensenada (CICESE), Ensenada, Baja California 22860, México; Departamento de Ciencias de la Computación, Centro de Investigación Científica y de Educación Superior de Ensenada (CICESE), Ensenada, Baja California 22860, México; Grupo de Medicina Molecular y Translacional (MeM&T), Facultad de Medicina, Universidad San Francisco de Quito (USFQ), Quito, Ecuador; Departamento de Ciencias de la Computación, Centro de Investigación Científica y de Educación Superior de Ensenada (CICESE), Ensenada, Baja California 22860, México

## Abstract

**Motivation:**

Antimicrobial peptides (AMPs) are promising molecules to treat infectious diseases caused by multi-drug resistance pathogens, some types of cancer, and other conditions. Computer-aided strategies are efficient tools for the high-throughput screening of AMPs.

**Results:**

This report highlights *StarPep Toolbox*, an open-source and user-friendly software to study the bioactive chemical space of AMPs using complex network-based representations, clustering, and similarity-searching models. The novelty of this research lies in the combination of network science and similarity-searching techniques, distinguishing it from conventional methods based on machine learning and other computational approaches. The network-based representation of the AMP chemical space presents promising opportunities for peptide drug repurposing, development, and optimization. This approach could serve as a baseline for the discovery of a new generation of therapeutics peptides.

**Availability and implementation:**

All underlying code and installation files are accessible through GitHub (https://github.com/Grupo-Medicina-Molecular-y-Traslacional/StarPep) under the Apache 2.0 license.

## 1 Introduction

Antimicrobial peptides (AMPs) are small molecules (generally ∼50 amino acids) presented as potential drugs to treat infectious diseases caused by multi-drug resistance pathogens (Browne *et al.* 2020), some types of cancers (Roudi *et al.* 2017), and other complex conditions (Mahlapuu *et al.* 2020). The efficiency of AMPs relies on their wide microbicidal and/or immunomodulating properties, the slow emergence of antimicrobial resistance, and their multifunctional activities (e.g. antibacterial, antiviral, antiparasitic, anticancer, etc.) (Browne *et al.* 2020). The FDA has approved more than 80 peptide-based drugs, and many more are in different phases of clinical trials (Usmani *et al.* 2017), making them potential therapeutic agents.

*In silico* peptide drug discovery relies on the availability of large, diverse, and non-redundant databases of AMPs, which have been developed over the last few decades (Browne *et al.* 2020). The growing number of databases holds a valuable source of knowledge, but a comparative study by some of the authors of this work argued that there is an overlap in content among existing databases due to the multiple functions of AMPs (Aguilera-Mendoza *et al.* 2015). Because of this overlapping, a representative set of 45 120 AMPs and their biological annotations are present in StarPep*DB* (Aguilera-Mendoza *et al.* 2019), a graph database that integrated peptides and their metadata from 40 databases. StarPep*DB* has been used to create alignment-free machine-learning classifiers to predict several AMP-related activities (Pinacho-Castellanos *et al.* 2021). This study introduces the StarPep toolbox software, which incorporates the *StarPepDB* to retrieve AMPs and employs functionalities grounded in network science and similarity searching to analyze these molecules. The software aims to explore the chemical space of AMPs and facilitate the discovery and repurposing of novel molecules for peptide drug design and optimization.

## 2 Software modules

**Figure 1.**
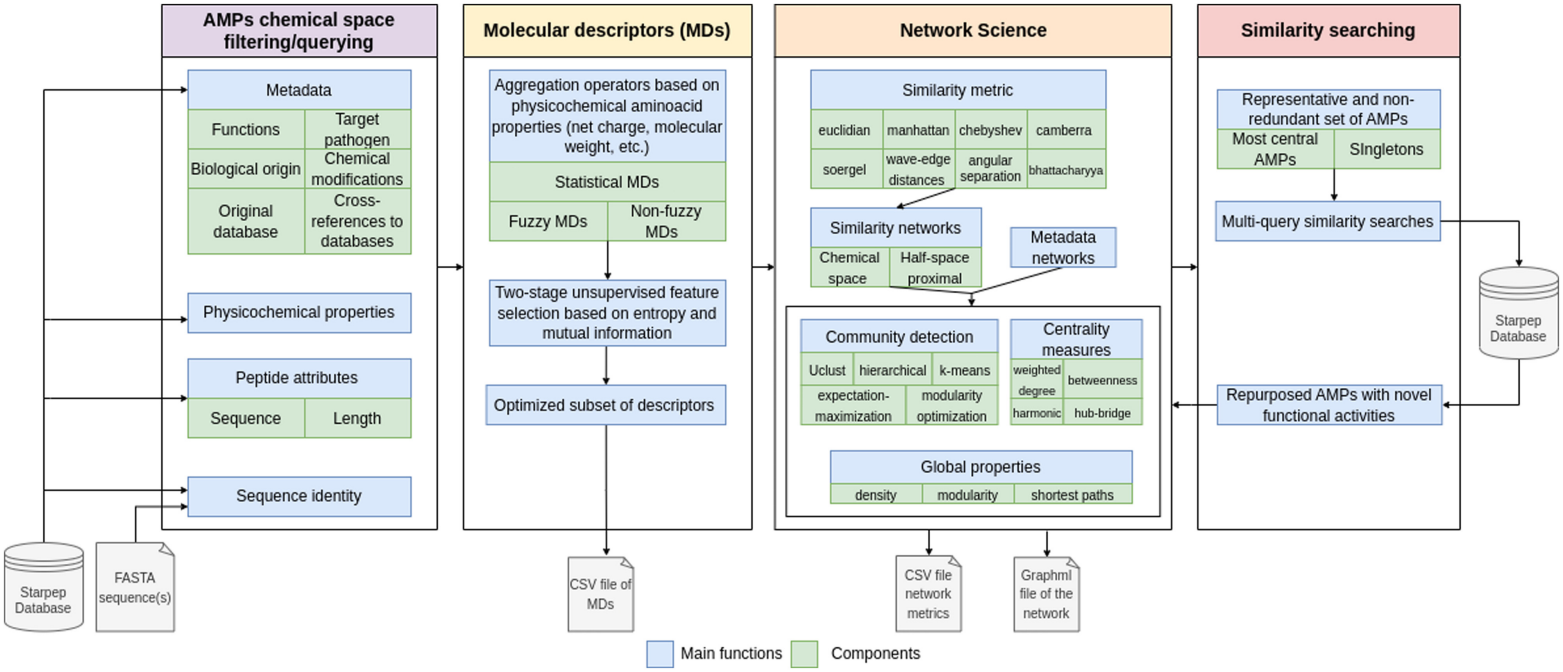
StarPep modules and components. The software has four main modules to load and filter AMPs, calculate MDs, create, and analyze networks, and develop mQSSMs.

Figure 1 shows the main modules and components of the *StarPep toolbox*. This software has four main modules: (i) load and filter AMPs from the internal StarPep*DB* or an external file (imported as a *FASTA* file), (ii) calculate optimized molecular descriptors (MDs) set to define a multidimensional descriptor space from the selected set of AMPs, (iii) create and visualize complex networks (*metadata* or *similarity* networks) for understanding hidden knowledge in data, and (iv) develop multi-query similarity-searching models (mQSSMs) to predict biological functions of AMPs. The functionalities of each module are explained in detail below.

### 2.1 AMPs chemical space filtering/querying

*StarPep toolbox* allows users to retrieve a subset of AMPs from the *StarPepDB* using their metadata (function, target pathogen, biological origin, chemical modifications, original database, and cross-referenced entries to Protein Data Bank, PubMed, and UniProt). It is also possible to find AMPs that share a given sequence identity above a threshold with single or multiple queries from the *StarPepDB* or an external sequence. Moreover, the software can create a non-redundant set of AMPs where any pair of sequences share no more than a given threshold of sequence identity. The user can choose the parameters to calculate the sequence identity (i.e. global, or local alignment algorithm, the substitution matrix, etc.). Finally, the software enables filtering out AMPs from the *StarPepDB* by peptide attributes (sequence or length), physicochemical properties, metadata node names, and PDB availability. The filtering module supports complex queries with AND/OR/NOT operators. Aguilera-Mendoza *et al.* (2019) provided a detailed explanation of this module, and further information is available in the online documentation of the software (https://grupo-medicina-molecular-y-traslacional.github.io/StarPep_doc/networks.html). In addition, specific use cases have been reported by Ayala-Ruano *et al.* (2022), Romero *et al.* (2022), Castillo-Mendieta *et al.* (2023), and Agüero-Chapin *et al.* (2023).

### 2.2 Molecular descriptors

*StarPep toolbox* integrates an option to compute protein/peptide sequence-based MDs. These MDs are calculated by applying statistical (e.g. measures of central tendency, and statistical dispersion), fuzzy (e.g. *Choquet* integral), and non-fuzzy (e.g. *GOWAWA*) aggregation operators on physicochemical amino acid properties (e.g. net charge, isoelectric point, molecular weight, etc.) [see SI1–4 in Aguilera-Mendoza *et al.* (2020)]. The proposed workflow in the *StarPep toolbox* takes peptide sequences as input to project all these *n* compounds into an *m*-dimensional space of hundreds of MDs. These sequence-based MDs showed better behavior (Aguilera-Mendoza *et al.* 2020) than the *iFeature* software (Chen *et al.* 2018). In this module, the set of initially calculated MDs can be reduced to find an optimized subset by applying a two-stage unsupervised feature selection pipeline, which is based on entropy and mutual information concepts (Aguilera-Mendoza *et al.* 2020). The optimized sets of MDs enable the computation of similarity indices, perform clustering analysis, and create correlation networks of AMPs; as explained in the next section. The MDs can be employed within the StarPep toolbox, or alternatively, exported as CSV files for external usage in various tasks, including the development of predictive models using machine-learning techniques. Thus, *StarPep toolbox* is a new software for calculating peptide/protein features that generalize most of the sequence indices families proposed to date.

### 2.3 Network science

*StarPep toolbox* supports the creation of three types of networks: (i) metadata (METNs), (ii) chemical space (CSNs), and (iii) half-space proximal (HSPNs). METNs are knowledge graphs where the edges represent semantic relationships (e.g. “is a,” “produced by,” “compiled in,” etc.) between the AMPs and their metadata [see Fig. 1 in Aguilera-Mendoza *et al.* (2019)]. CSNs and HSPNs are correlation and weighted networks, where the weights are the similarity values, and two nodes are connected by an edge if the similarity coefficient is greater than or equal to a given cutoff. The software provides different metrics to define the similarity between AMPs (Euclidean, Manhattan, Chebyshev, Camberra, Soergel, Bhattacharyya, Angular separation, and Wave-edge distances). A recent study found that the selection of similarity metrics for the construction of hemolytic peptide networks significantly affected the results (Castillo-Mendieta *et al.* 2023). CSNs and HSPNs differ in their building methods, with CSNs typically having more edges than HSPNs (Aguilera-Mendoza *et al.* 2020). HSPNs have been demonstrated to yield better outcomes than CSNs in prior research (Ayala-Ruano *et al.* 2022, Romero *et al.* 2022). This finding could be attributed to the fact that HSPNs are constructed without searching for an optimal similarity threshold, as they rely on the application of the half-space proximal test (Chavez *et al.* 2006).

After creating the similarity networks, the software provides global properties (e.g. density, modularity, etc.) and calculates centrality measures (weighted degree, betweenness, harmonic, and hub-bridge) that give a notion of the importance of nodes (peptides) in a complex network. Moreover, the program includes community detection algorithms (modularity optimization, hierarchical, *k*-means, expectation–maximization, and Uclust), for detecting potential AMPs families with similar MD profiles. Zooming into the detected clusters helps to identify central nodes representing relevant AMPs within communities. Moreover, the clustering analysis offers the possibility of identifying singleton (atypical) nodes, representing isolated peptides with distinct characteristics from the grouped ones. These important nodes facilitate the identification of shared motifs among potential AMP families organized into distinct communities. Previous research identified such motifs for different AMPs, including antiparasitic (Ayala-Ruano *et al.* 2022), tumor-homing (Romero *et al.* 2022), hemolytic (Castillo-Mendieta *et al.* 2023), and antibiofilm peptides (Agüero-Chapin *et al.* 2023). In addition, these nodes serve as the basis for the subsequent creation of mQSSMs, as detailed in the next section. There are also options to get a non-redundant set of nodes based on a centrality measure. This can be carried out by using the *scaffold extraction* plugin and calculating the shortest path between two nodes with the *shortest path* plugin. The software presents an additional feature that enables the overlapping of external sequences onto a network, finding the *k*-nearest neighbors and facilitating the prediction of novel activities leveraging the information from the neighborhood. The visualization of networks can be customized by changing the color and size of nodes according to node-level parameters (e.g. centrality measures, communities, etc.), or by applying different layouts that spatialize and rearrange nodes. Furthermore, the networks can be represented with the principal components, which are linear combinations of the MDs calculated with the software. The online documentation of the software presents a comprehensive description of most of the features from this section.

### 2.4 Similarity searching

After applying centrality measures and community detection algorithms in the CSNs and HSPNs, the software gives a representative and non-redundant set of AMPs. These AMPs comprise the most important nodes by each community and the singletons, representing a sample of the whole chemical space. This set can be the input to carry out multi-query similarity searches against the *StarPepDB* [see Fig. 7 in Agüero-Chapin *et al.* (2022)]. The mQSSMs are untrained one-class models that do not require datasets for non-AMPs, which is an advantage because these negatively labeled datasets are normally created artificially and reduce the classification capacity of the algorithms (Sidorczuk *et al.* 2022). The mQSSMs can lead to the repurposing of AMPs with novel functional activities. Prior research has demonstrated that mQSSMs can outperform *state-of-the-art* methods for predicting antiparasitic (Ayala-Ruano *et al.* 2022) and tumor-homing peptides (Romero *et al.* 2022).

## 3 Implementation

*StarPep toolbox* is an open-source desktop application developed in Java, using Java Development Kit (JDK) 8 and integrating functionalities from other Java-based open-source projects. For instance, the graphical user interface was built on top of Gephi (Bastian *et al.* 2009), which in turn is based on the *NetBeans Platform* (https://platform.netbeans.org/). The graph database structure was implemented in *Neo4j* (https://neo4j.com/). The visualization engine and some network-based metrics and methods were imported from *Gephi* (Bastian *et al.* 2009). The sequence alignment algorithms were implemented using *BioJava* (Lafita *et al.* 2019). The 3D structures attached to peptide nodes (PDB metadata) may be displayed inside the software tool by using *Jmol* (http://www.jmol.org/).

## 4 Conclusions and future perspectives

*StarPep toolbox* is an open-source and user-friendly software to explore the AMP chemical space by querying, filtering, visualizing, and analyzing network-based representations. This application opens many possibilities for peptide drug repurposing, design, and optimization. We plan to add new functionalities to the software, such as new centrality metrics, more global and node-specific network properties, a module for structural motifs searching, and a web version of the StarPep*DB* with some tools of the software.

## Data Availability

The *StarPep toolbox* software and the installation files are freely available at https://github.com/Grupo-Medicina-Molecular-y-Traslacional/StarPep and the online documentation at https://grupo-medicina-molecular-y-traslacional.github.io/StarPep_doc.
